# Determining the Effectiveness of a New Device for Hand Therapy (The FEPSim Device): Feasibility Protocol for a Randomized Controlled Trial Study

**DOI:** 10.2196/22145

**Published:** 2021-05-27

**Authors:** Antonio Miguel-Cruz Sr, Christine Guptill, Geoffrey Gregson, Anna-Maria Ladurner, Cindy Holmes, Daniel Yeung, Justine Siebert, Gwen Dziwenko, Adriana Ríos Rincón

**Affiliations:** 1 Department of Occupational Therapy Faculty of Rehabilitation Medicine University of Alberta Edmonton, AB Canada; 2 Glenrose Rehabilitation Research Innovation and Technology Glenrose Rehabilitation Hospital Edmonton, AB Canada; 3 Royal Alexandra Hospital Edmonton, AB Canada; 4 Glenrose Rehabilitation Hospital Edmonton, AB Canada

**Keywords:** technology assessment, hand therapy, technology for rehabilitation, clinical engineering, biomedical engineering

## Abstract

**Background:**

Impairments of the forearm, wrist, and hand affect a sizable proportion of individuals and impose a significant economic burden on health care systems. FEPSim is a medical device for hand and wrist rehabilitation. The FEPSim device could be part of the standard of care for upper extremity rehabilitation during therapeutic activities to increase range of motion, dexterity, and strength. FEPSim has not yet been tested in a health care setting; therefore, a trial of the effectiveness of FEPSim in upper extremity rehabilitation is warranted.

**Objective:**

This study aims to assess the feasibility of conducting a definitive trial in terms of recruitment, eligibility criteria, the type and number of diagnoses included, the length and dosage of the intervention, and data collection methods. This study also aims to gather clinical and statistical information as well as information related to the cost and usability, which allows for an economic evaluation of the device.

**Methods:**

The trial will use a randomized controlled design comprising 47 intervention participants and 47 control group participants. Participants will be adults (age≥18 years) attending outpatient rehabilitation with limitations in their forearm, wrist, or hand function due to distal radial or ulnar fractures, stroke, or osteoarthritis. This study’s primary outcome variables are related to patients’ range of motion and strength, specifically active and passive wrist flexion and extension range of motion; active and passive forearm pronation and supination range of motion; grip strength; and pinch strength. The secondary outcome variables are related to patients’ perceived wrist pain and disability in activities of daily living. The patients’ perceived wrist pain and disability in activities of daily living will be measured using the patient-rated wrist evaluation questionnaire. The control group will receive the standard of care at each of the 2 hospital facilities (Glenrose Rehabilitation and Royal Alexandra Hospitals). The intervention group will receive the same standard of care as the control group at each facility and will use the FEPSim device for therapeutic activities to increase strength, range of motion, resistance, and dexterity. All the participants will be assessed at baseline (week 0); weeks 2, 4, and 8; and postintervention (week 10).

**Results:**

The FEPSim study was launched in April 2020. This study is currently on hold because of the global COVID-19 pandemic. The recruitment process is expected to resume by September 2020, and the primary impact analysis is expected to be conducted by December 2020.

**Conclusions:**

This study will provide valuable information on the measurement of comparative intervention effects, technology acceptance by hand therapists, and how associated treatment and product costs will contribute to the evidence planning process, which will be crucial for the future adoption of FEPSim.

**Trial Registration:**

International Standard Randomized Controlled Trial Number Registry ISRCTN13656014; https://www.isrctn.com/ISRCTN13656014

**International Registered Report Identifier (IRRID):**

PRR1-10.2196/22145

## Introduction

### Background and Rationale

People affected by musculoskeletal disorders (MSDs) of the forearm and wrist, such as fractures (including those exacerbated by osteoporosis) and osteoarthritis, as well as people who have had a stroke will experience impairments of the upper limbs [1]. Impairments of the upper limbs affect functioning in everyday life and are correlated with a low quality of life [2]. Impairments of the forearm, wrist, and hand represent a health-related problem that affects a sizable proportion of individuals and impose a significant economic burden for health care systems. For example, in Alberta, Canada, by 2017, forearm fractures accounted for 17,031 cases with an incidence rate of 441 (new cases), the prevalence of osteoporosis accounted for 174,481 cases with an incidence rate of 18,603 (new cases); 21.00% (36,641/174,481) of people who have osteoporosis will have a fracture (eg, wrist fractures) [3]; the prevalence of different types of strokes accounted for 5277 cases with an incidence rate of 115 (new cases); 49.54% (2614/5277) of people who had a stroke also experienced an upper limb impairment; and the prevalence of osteoarthritis accounted for 449,561 cases with an incidence rate of 34,479 (new cases). The economic burden on Alberta’s health system as a result of caring for impairments of the forearm, wrist, and hand is significant and is expected to increase along with the projected increase in the age and size of the population. By 2017, the average cost of hospital inpatient care in Alberta totaled CAD $18,642,407 (US $15,287,926.24), an increase of 7.7% compared with the average cost in 2016 [4].

FEPSim, developed by Karma Machining & Manufacturing Ltd, is a medical device for hand and wrist rehabilitation. The FEPSim device could be part of the standard of care for upper extremity rehabilitation during therapeutic activities designed to increase range of motion, dexterity, and strength. These activities include controlled movements, strengthening, and exercises for retraining different grasp patterns that are used for activities of daily living and work tasks. Grading these activities is important to achieve therapeutic objectives and measure improvement. However, the equipment that is usually available in clinical settings does not allow therapists to ascertain their patients’ range of motion or the strength of their arms and hands (eg, wrist pronation or supination and flexion or extension) during functional hand movements and grasp patterns. FEPSim is a medical device that was developed for upper extremity rehabilitation and is used to strengthen the hand and wrist with movements such as wrist flexion and extension; hand and forearm pronation and supination; and different grasp patterns such as power grasp, spherical grasp, lateral grip, and disk grasp. FEPSim can be adjusted according to the patient’s capabilities during the rehabilitation process, thus allowing the therapist to grade the activities in terms of resistance and repetitions of any given exercise. FEPSim also allows the therapist to ascertain the patient’s strength and the degrees of range of motion that are achieved during active movements of the hand or forearm.

The FEPSim device appears to have potential advantages over current technologies. Hand therapy devices fall into 1 of the following 2 categories:

1. Low-cost and portable devices (average price: CAD $127.07 [US $104.16]) designed to offer specific hand therapies (eg, pronation or supination), such as *Rolyan Pronation/Supination* (CAD $188.57 [US $154.58]), or strengthen the forearm, such as *The Pronator* (CAD $108.64 [US $ 89.05]; a price list is available at Performance Health Trademarks [5]). The main disadvantage of these devices is that important measurements during hand therapy activities cannot be taken directly from them, and they are not adjustable.
2. High-cost (portable and nonportable devices) commercial electromechanical devices designed to simulate the basic motions are required by the upper extremities in most occupations and to conduct hand therapy. This is the case for the Baltimore Therapeutic Equipment (BTE) work simulator and SaeboReJoyce [6]. For example, the estimated price of the BTE is between CAD $60,012.80 (US $49,193.99) and CAD $113,357.52 (US $92,921.99; email communication with the BTE Senior National Sales Representative) [7], whereas the SaeboReJoyce costs CAD $17,350.68 (US $14,222.79) [8]. The high cost of the BTE and SaeboReJoyce means that these devices are simply not affordable in many health care settings; thus, the FEPSim device could be a more affordable alternative for hand therapy purposes. The current prototype selling price of an FEPSim device is approximately CAD $6000 (US $4920; unit cost; eg, cost of goods sold). After evaluation and market analysis, to determine the scale of production, the FEPSim device should retail for less than CAD $1300 (US $ 1065.64) if the company adopts injection molding and brings in more purchased parts, with future models bringing costs down further. In addition, FEPSim has other potential advantages, such as compactness, portability, and ease of use.

Robots could be an alternative to assist in the rehabilitation process of the hand. However, these robotic systems are not yet available in hand therapy settings. A recent survey that aimed to examine patents and developments for hand rehabilitation robots found 28 systems, among which only 1, the Hand Exoskeleton Rehabilitation Robot, was designed to provide continuous passive motion (ie, the robot executes the motion of the patient’s upper limbs) as well as active independent movements (ie, the patient performs a motion according to his or her own ability) to the fingers and the thumb [9]. In another review, only 2 robotic devices were found for active independent movement of the hand [10]. All these robotic devices for hand rehabilitation were still in the prototype design phase, which corresponds to a technology readiness scale lower than 5 [11]; therefore, they have not yet been tested in real rehabilitation contexts.

### Hand Therapy: Existing Knowledge

The standard of care for rehabilitation for impairments of the forearm, wrist, and hand includes a combination of modalities and techniques such as immobilization, management of scar tissue, sensory modification, edema management, and therapeutic activities to increase the range of motion; dexterity and strength; and, ultimately, hand function [2]. The current evidence for the therapeutic standard of care activities depends on the medical condition and the strategies or modalities used to achieve therapeutic outcomes. In general, Roll and Hardison [2] found that for all MSDs, the strongest evidence for occupational therapy interventions supports postsurgical early active motion protocols and splinting for various conditions. However, few studies have shown significant differences in long-term outcomes among the compared interventions. For osteoarthritis, in particular, there is limited evidence that education and exercise can help patients regain function and reduce pain, whereas the evidence for the use of splinting for the same purposes is mixed. A systematic literature review summarized the outcomes of 26 studies for rehabilitation after distal radial fractures, finding that all the studies had low-quality designs and no clinically essential outcome differences among the modalities implemented in the interventions. The outcomes were categorized as functional (eg, range of motion, pain, grip strength, and activities of daily living), clinical (eg, residual soft tissue swelling), and resources (eg, number of outpatient attendances). The authors also found that, despite a lack of evidence for greater effects regarding long-term (3 months) goals, early occupational therapy led to more short-term improvements in gripping, pinching, and range of motion [12].

Strokes also affect hand function. The interventions that have been effective in managing spasticity are constraint-induced movement therapy, mirror therapy, and functional skill retraining. Furthermore, the recommended activities are passive range of motion (PROM) and active range of motion (AROM) activities, along with movements and functional activities with high levels of repetition [13]. Another review stated that a patient with a neurological condition that affects his or her hand movement (eg, stroke) needs to repeat a motion 300 to 400 times to learn a movement, but in current therapy sessions, the standard is closer to 30 repetitions. In a clinical setting, the recommended dosage would be a 60-minute session, 3 times a week for 6 weeks, where the use of technology can help achieve more repetitions in a shorter period of time [14]. Burns may also affect hand function. In general, the standard of care includes edema management, splinting, patient caregiver education, range of motion and strengthening, scar management, and retraining in activities of daily living [15]. We did not find any research that examined the evidence for these modalities. Finally, a literature review about the cost-effectiveness of physiotherapy interventions found only 2 studies about hand rehabilitation in neurological conditions. None of these studies reported any significant cost-effectiveness between the interventions under study. However, one study in which a high-tech device (ie, a robotic system) was used to support hand therapy showed that a group that used robot intervention used less health care, which reduced the overall cost [16].

### Evaluation Objectives and Research Questions

FEPSim has not yet been tested in a health care setting; therefore, a trial of the effectiveness of FEPSim in upper extremity rehabilitation is warranted. The primary objective of this study is to assess the feasibility of conducting a definitive trial in terms of recruitment, eligibility criteria, the type and number of diagnoses included, the length and dosage of the intervention, and the data collection methods. This study also aims to gather clinical and statistical information as well as information related to the costs and usability (adoption) of the new technology used in this study. Thus, this study has 6 secondary objectives:

1. To explore the clinical effectiveness of adding the FEPSim device to the standard of care for patients with injuries and clinical conditions of the forearm, wrist, and hand.
2. To assess the outcome measures for measuring changes in the dependent variables.
3. To gather and synthesize the data, from which the sample size of a definitive randomized controlled trial (RCT) can be estimated.
4. To measure the key outcome domains (for completion rates, missing data, estimates, variances, and 95% CI for the differences between the intervention and control groups) for patients with injuries and clinical conditions of the forearm, wrist, and hand.
5. To examine the total and component costs associated with the FEPSim device and with standard of care interventions for patients with injuries and clinical conditions of the forearm, wrist, and hand from an institutional perspective (ie, hospitals).
6. To investigate the usability of the FEPSim device by therapists.

## Methods

### Study Design

This study will use a multimethod research design. The *Methods* section will be presented with regard to the objectives of the study.

### Primary Objective and Secondary Objectives 1 to 4: Research Design

The feasibility parallel-group RCT will follow the CONSORT (Consolidated Standards of Reporting Trials) guidelines for randomized feasibility studies [17]. The experimental group will receive an intervention consisting of sessions with the FEPSim device plus the standard of care, whereas the control group will only receive the standard of care for hand therapy.

### Secondary Objective 5: Study Design

For the economic evaluation component of this study, we will follow the Consolidated Health Economic Evaluation Reporting Standards [18] and Guidelines for the Economic Evaluation of Health Technologies: Canada [19].

### Secondary Objective 6: Study Design

For the usability component of this study, we will follow a qualitative description design [20].

### Study Setting

The study will be conducted in 2 health care facilities: the Royal Alexandra Hospital Outpatient Clinic and the Glenrose Rehabilitation Hospital Specialized Rehabilitation Outpatient Program Hand Class. Both institutions are located in Edmonton, Alberta, Canada.

### Eligibility Criteria

#### Inclusion Criteria: Participants (Patients)

This study will include outpatient adults (≥18 years) with limitations in their forearm, wrist, or hand function due to distal radial or ulnar fractures, stroke, or osteoarthritis (eg, patients who have undergone a wrist salvage procedure).

#### Inclusion Criteria: Participants (Therapists)

This study will include hand therapists from hand therapy services who have used the FEPSim device.

#### Exclusion Criteria: Participants (Patients)

Outpatients will not be included in our study if they (1) have chronic regional pain syndrome as these participants’ baseline measurements would differ too much and they would experience abnormal pain responses; (2) report subjective or patient limitations that prevent them from participating (eg, excessive pain and edema); (3) are unable to participate in the program (outpatient hand clinic) twice a week (eg, transportation and limited buy-in); (4) have limitations in their reading and listening comprehension of the English language that prevent them from understanding the patient-rated wrist evaluation (PRWE) questionnaire; or (5) have limitations in following instructions due to a severe cognitive impairment.

### Interventions

The eligible participants will be randomly assigned in a 1:1 ratio to either the experimental group or the control group.

#### Control Group

This group will receive the standard of care at each hospital, which consists of immobilization for 7 to 8 weeks (for fractures) after the time of the injury or surgery, followed by hand therapy sessions for 10 weeks to manage scar tissue, sensory modifications, and edema as well as therapeutic activities to increase strength, range of motion, and dexterity. These therapeutic activities will be carried out using the equipment and materials available at each hospital, including weights and elastic or gripping equipment and materials. The sessions’ length and frequency will depend on the patients’ needs and diagnoses. The length of each session will be between 30 minutes and 45 minutes, and they will be carried out once or twice per week.

#### Experimental (Intervention) Group

This group will receive the same standard of care as the control group at each hospital, which consists of immobilization for 7 to 8 weeks after the time of the injury or surgery, followed by hand therapy sessions for 10 weeks to manage scar tissue, sensory alterations, and edema. The experimental group will use the FEPSim device for therapeutic activities to increase strength, range of motion, and dexterity. For this group, the sessions’ length and frequency will depend on the patients’ needs and diagnoses. The length of each session will be between 30 minutes and 45 minutes, and they will be carried out once or twice per week.

### Outcome Variables

#### Primary Objective and Secondary Objectives 1 to 4: Primary Outcome Variables

The retention rates and intervention compliance will be calculated for the primary objective. The retention rates will be calculated according to the participants’ discontinuation of the interventions and their absence at the posttest at week 10. Intervention compliance means all of the hand rehabilitation sessions are completed by each group [21]. This study’s primary outcome variables for secondary objectives 1 to 4 are related to range of motion and strength: (1) AROM and PROM of wrist flexion and extension and forearm pronation and supination, (2) grip strength, and (3) pinch strength.

#### Primary Objective and Secondary Objectives 1 to 4: Secondary Outcome Variables

The secondary outcome variables are the patients’ perceived wrist pain and disability in activities of daily living.

#### Primary Objective and Secondary Objectives 1 to 4: Confounding Variables

The confounding effects of the participants’ age, gender, and medical condition (distal radial or ulnar fractures, stroke, or osteoarthritis); whether the participants are taking any pain medication; what activities the participants perform at home or work; and the therapist providing the intervention will be determined.

#### Secondary Objective 5: Estimating the Costs and Resource Use

This study aims to ascertain which factors result in differences in costs or outcomes when comparing the standard of care with the FEPSim device. The estimated costs of the interventions will be from an institutional perspective, that is, the Glenrose Rehabilitation and Royal Alexandra Hospitals. To estimate the resource use and costs, we will use a single study–based economic evaluation approach (patient-level data). The costs will include resources related to the following categories [19]: cost of the time spent by the human resources (hand therapists and support personnel, if applicable) involved in each of the interventions; this time will be converted to cost based on the average salary for each level of staff. It will also include the time human resources spend on the training sessions, learning to use the FEPSim device (ie, training sessions), and seeking technical support (in the intervention group).

#### Secondary Objective 6

We will determine the usability and technology acceptance of the FEPSim device based on the Unified Theory of Acceptance and Use of Technology constructs, that is, performance expectancy (FEPSim was useful); effort expectancy (learning to use FEPSim was easy); facilitating condition (using FEPSim was well suited to my needs); social influence (people who are important to me think that I should use FEPSim); behavioral intention to use FEPSim (I plan to use FEPSim in the near future); and actual use of FEPSim, if applicable [22].

### Participant Timeline

**Figure 1:**
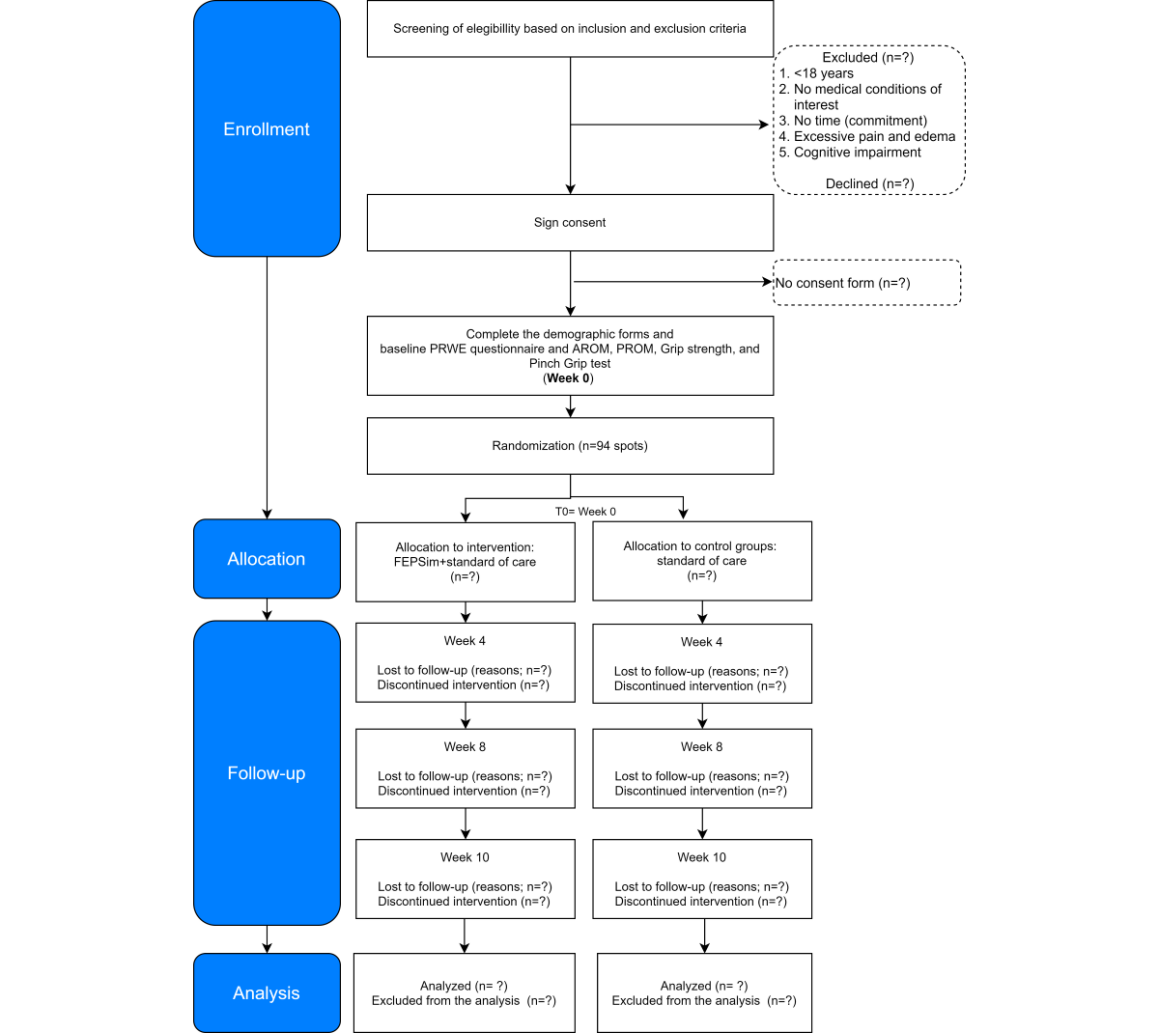
Flow of participants. AROM: active range of motion; PROM: passive range of motion; PRWE: patient-rated wrist evaluation questionnaire; T0: data collection point 1.

**Figure 2:**
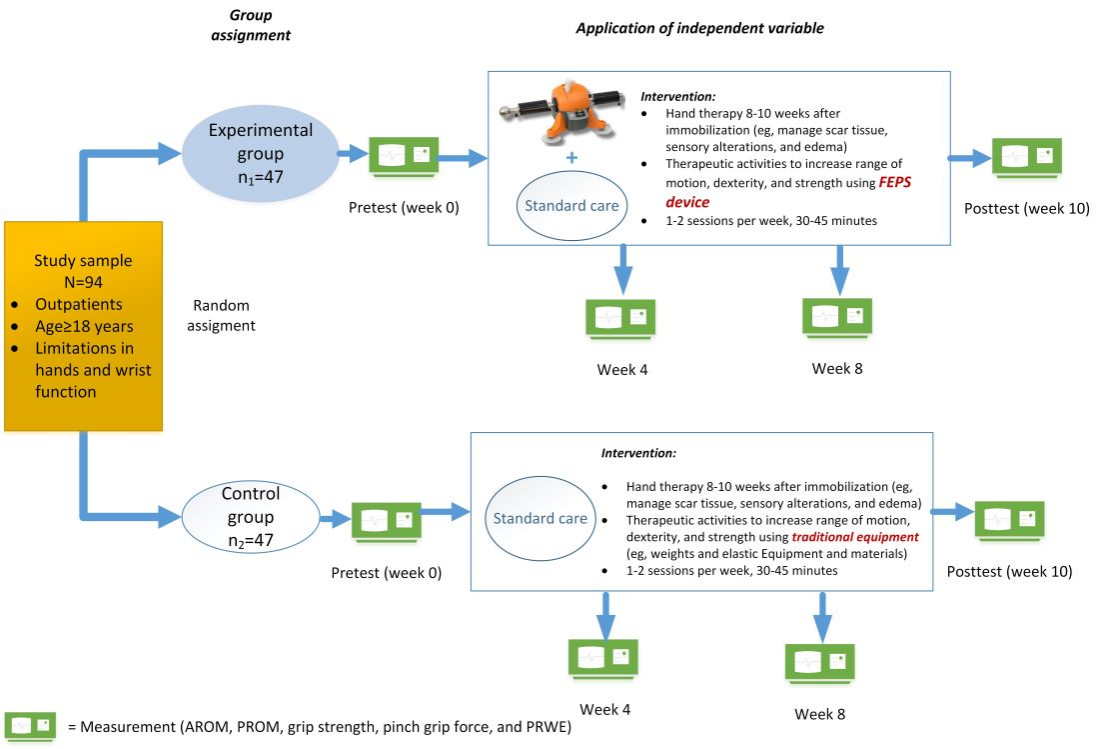
Study design schema (primary objective and secondary objectives 1-4). AROM: active range of motion; PROM: passive range of motion; PRWE: patient-rated wrist evaluation questionnaire.

This feasibility trial consists of a 10-week intervention treatment phase; this study does not have a follow-up phase. The total trial data collection period will be 9 months. As shown in Figures 1 and 2 and in Table 1, measurements will be taken at 4 points in time for each group: at baseline (week 0); during weeks 2, 4, and 8; and after the intervention (week 10).

**Table 1:** Key variables and measurements.

Variables			Participants		Measurement		T_0_^a^		T_1_^b^		T_2_^c^		T_3_^d^
**Primary outcome**													
	AROM^e^	Patients		Goniometer		✓		✓		✓		✓	
	PROM^f^	Patients		Goniometer		✓		✓		✓		✓	
	Grip strength	Patients		Dynamometer		✓		✓		✓		✓	
	Pinch grip	Patients		Pinch meter		✓		✓		✓		✓	
**Secondary outcomes**													
	Perceived wrist pain and disability during daily life activities	Patients		PRWE^g^ questionnaire		✓		✓		✓		✓	
**Measures of usability and technology acceptance**													
	Performance expectancy	Therapists		Semistructured interview								✓	
	Effort expectancy	Therapists		Semistructured interview								✓	
	Facilitating condition	Therapists		Semistructured interview								✓	
	Social influence	Therapists		Semistructured interview								✓	
	Behavioral intention to use the FEPSim	Therapists		Semistructured interview								✓	
	Actual use of the FEPSim	Therapists		Semistructured interview								✓	
**Measurements of economic evaluation**													
	Costs	N/A^h^		Questionnaire for costs associated with use of the FEPSim and provision of the intervention		✓						✓	
**Covariates**													
	Demographic variables	Patients		Self-report assessment questionnaire		✓							
	Medical condition (distal radial or ulnar fractures, stroke, or osteoarthritis)	Patients		Therapist records		✓							
	Whether the participant is taking any pain medication	Patients		Self-report assessment questionnaire		✓		✓		✓		✓	
	Activities the participant preforms at home or work	Patients		Self-report assessment questionnaire		✓		✓		✓		✓	

^a^T_0_: data collection point 1.

^b^T_1_: data collection point 2.

^c^T_2_: data collection point 3.

^d^T_3_: data collection point 4.

^e^AROM: active range of motion.

^f^PROM: passive range of motion.

^g^PRWE: patient-rated wrist evaluation.

^h^N/A: not applicable.

### Sample Size

#### Primary Objective and Secondary Objectives 1 to 4

As this is a feasibility study, a sample size calculation is not required [17]. However, we can estimate the number of participants we will be able to recruit during the data collection period. According to the clinical partners at the 2 hospitals involved in this study, approximately 167 patients who potentially meet the inclusion criteria will be admitted (accessible population). We aim to recruit 47 participants for each group, for a total sample size of 94 participants, to compensate for a 30% dropout rate. Thus, the recruitment of 94 participants represents 56.3 % (94/167) of the participation rate. This number is based on our ability to detect a small effect size (Cohen *d*=0.25) with 80% power and an α of .05 (two-sided). The sample size calculations were estimated using G*Power software version 3.1.9.4 (Universitat Kiel) [23].

#### Secondary Objective 6

Ten hand therapists from the hand therapy services who have used the FEPSim device will be recruited. Individual interviews will be conducted until either redundancy or theoretical saturation of the data has been achieved [24].

### Recruitment

#### Participants: Patients

An invitation to participate will be posted in several locations at the Glenrose Rehabilitation and Royal Alexandra Hospitals. Hand therapists at both the hospitals will support the recruitment strategies, including the provision of information sessions and one-on-one conversations with potential participants. The first contact with a potential participant will be made through one of the hand therapists not involved in the research team. Therapists who are already involved in the clinical care of the patients will then determine the individuals’ willingness to be approached by the hand therapist researcher regarding participation and obtain their consent for the study.

#### Participants: Therapists

The study coordinator will send out an invitation to participate via email to potential participating therapists, and posters will be put up in the therapists’ staff rooms at the Glenrose Rehabilitation and Royal Alexandra Hospitals.

### Allocation: Sequence Generation

Probability sampling stratified by medical condition (wrist fractures, acquired brain injuries, burns, or osteoarthritis) will be used. Random sequence generation will be prepared in advance by a research team member (AMC Sr) on an Excel file spreadsheet (RAND function) using permuted block randomization with a block size of 4 and a ratio of 1:1.

### Allocation: Concealment Mechanism

Allocation concealment will be ensured, as we will not release the randomization code until the patients have been recruited in the trial and all the baseline measurements have been completed.

### Allocation: Implementation

If a potential participant meets the inclusion criteria, the hand therapist researchers (CH, DY, GD, and JS) will ask the study coordinator (AML) to check whether a place is available in the study for that participant in a given strata (ie, medical condition). If a place is available, then one of the therapist researchers (CH or DY) or another therapist involved in the recruitment process (JS or GD) will invite the participant to participate in the study, explain the study to him or her, and ask him or her to sign the consent form. If a potential participant is assigned to a particular therapist researcher (CH, DY, JS, or GD), this therapist will not invite this participant to participate in the study. Instead, a secondary therapist researcher will do so. As a result, the freedom to decline will not be compromised. Once the participants or their substitute decision makers have signed the consent form and given their assent, the therapist researchers (CH or DY) will inform the study coordinator (AML). The study coordinator (AML) will allocate each participant to one arm of the trial according to the allocation protocol and assign a code. This code will be provided to the therapist researchers (CH or DY) and research assistants (RAs).

### Blinding (Masking)

The assessments of range of motion and strength measurements and the PRWE questionnaire will be conducted by RAs blinded to the treatment allocation. Due to the nature of the intervention, neither the participants nor the therapist can be blinded to the treatment allocation, but they are strongly encouraged not to disclose the participants’ allocation status during the assessments. An RA will enter the data onto a computer on separate datasheets, and a senior RA will conduct the data analysis under the supervision of the principal investigators (AMC Sr and AMRR).

### Data Collection Methods

#### Primary Objective and Secondary Objectives 1 to 4: Primary Outcome Variables

The AROM and PROM of the wrist extension or flexion, radial and ulnar deviation, and pronation and supination will be measured using a goniometer (Baseline 360-degree, 12-inch clear plastic goniometer); the grip strength will be measured using a dynamometer (Baseline Lite hydraulic, 200 lb); and the pinch strength will be measured using a pinch gauge or pinch meter (Jamar pinch gauges).

#### Primary Objective and Secondary Objectives 1 to 4: Secondary Outcome Variables

The patients’ perceived wrist pain and disability in activities of daily living will be measured using the PRWE [25]. The PRWE is a 15-item questionnaire that assesses 3 components: pain, function during specific activities of daily living, and function during usual activities (personal care, household work, work, and recreational activities). Studies have found this questionnaire to be a valid and reliable assessment tool for evaluating patient-based pain and disability levels in routine clinical practice [26].

All hand therapy sessions for both groups will be conducted in the hand therapy area at the Glenrose Rehabilitation and Royal Alexandra Hospitals by the staff hand therapists. The length and frequency of the sessions for the participants in both groups will be recorded by the therapists. The RAs will measure the AROM, PROM, grip strength, and pinch strength. They will also administer the PRWE to all the participants before (week 0) and after the intervention (week 10) and during weeks 2, 4, and 8. The total time taken to administer the measures will be approximately 60 minutes. The RAs will be trained in the administration of the outcome measurements and adequate use of the assessment tools (eg, dynamometer, goniometer, and pinch meter) by experienced hand therapists.

#### Secondary Objective 5: Estimating the Costs and Resource Use

To estimate the resource use and costs, we will use a single study–based economic evaluation approach (patient-level data). The costs will include resources related to the following categories: (1) the time human resources spend on the sessions will be monitored during the study, whereas the information on the average salaries of the human resources will be taken from the records at each hospital; (2) the capital cost of the equipment used in each intervention and depreciation of the equipment will be calculated using the straight-line depreciation approach; (3) therapeutic supplies (eg, weights and elastic or squeezing supplies); (4) consumables (eg, bandages and sanitization wipes); (5) cost of sterilization, if needed; (6) allowance costs (if any); and (7) maintenance costs (eg, calibration, preventive, and corrective maintenance of equipment). The information on the costs will be taken from the financial records at each hospital. In addition, we will conduct face-to-face interviews with the financial departments at both hospitals to identify the cost components and costing method.

#### Secondary Objective 6: Usability and Technology Acceptance

Once the data collection for the primary objective is completed, semistructured interviews (topic guided) will be conducted with the hand therapists who have agreed to participate and signed a consent form. The interviews with the therapists who used the FEPSim device during their interventions will be audiotaped for later analysis by the team members. The interviews will be conducted by one RA. To ensure anonymity, the therapists’ responses will not be connected to their identities.

### Data Analyses

#### Primary Objective and Secondary Objectives 1 to 4

Data analyses will be conducted using the intention-to-treat principle. Complete case analysis will be the primary method for dealing with missing data equal to or more than 10%. This is the most common method for dealing with missing data and reducing the risk of bias [27]. For missing data of less than 10%, we will use a simple imputation method by replacing the missing data with the average participants in the same strata (ie, medical condition). The analyses will focus on descriptive statistics and CI estimation rather than formal hypothesis testing [28]. Descriptive statistics will be used to characterize the groups at the pretest and posttest as well as during weeks 2, 4, and 8. The outcome variables as well as other data such as the length and dosage of each intervention will be summarized as n (%), mean (SD), or median (IQR), as appropriate. The retention rates, intervention compliance, and missing data will be summarized for outcomes related to the secondary objectives, which, together with the estimates, will assist in calculating the sample size for a definitive trial. As this study aims to assess the feasibility of conducting a definitive trial, clinical effectiveness is a secondary objective (ie, the study’s proof-of-concept element). In this regard, comparisons of the outcome variables at the pretest (week 0) and posttest (week 10) within the groups will be performed using a paired two-tailed *t* test (or a Wilcoxon signed-rank test if the data are not normally distributed) for AROM, PROM, grip strength, and pinch grip force and a Wilcoxon signed-rank test for the PRWE. Comparisons between the groups at the pretest and posttest will be performed using a *t* test (or a Mann-Whitney *U* test if the data are not normally distributed) for AROM, PROM, and grip strength and a Mann-Whitney *U* test for the PRWE. With our sample size, there would be a power of 0.8 to detect a medium effect size of Cohen *d*=0.25. However, we are aware that our study might be underpowered due to the sample size. If no statistically significant differences are found, our results will be classified as inconclusive rather than negative. We will report 95% CIs and interpret the level of uncertainty based on them [28]. In addition, we will explore the effects of the covariates using general linear models or multilevel mixed models when appropriate. All the α levels of significance will be set at *P*≤.05 (two-tailed).

#### Secondary Objective 5: Estimating the Costs and Resource Use

For each intervention, descriptive statistics will be used to characterize the categories of the estimated costs (ie, mean [SD] or median [IQR] as appropriate). In this study, missing data costs will be replaced by means or medians, as appropriate. We will quantify uncertainties by reporting the differences between the means and 95% CIs for the categories of the comparator groups’ estimated costs and then interpret the levels of uncertainty based on these [28] and by conducting statistical tests (ie, a *t* test or Mann-Whitney *U,* test as appropriate), while looking for differences in each category of estimated costs between the comparator groups. All α levels of significance will be set at *P*≤.05 (two-tailed).

#### Secondary Objective 6: Usability and Technology Acceptance

The audiotapes will be transcribed, and content analysis will be performed. The content analysis will be data driven. The data codes will be generated inductively by the collected data. Along with coding, a small number of themes or categories will be generated. The analyses will be performed by an RA, and agreement in the interpretations will be achieved through a discussion between the research team members. The validity of the interpretations will be discussed with and agreed upon by every member of the research team.

Quantitative analyses will be conducted using the SPSS version 27.0, and qualitative analyses will be conducted using NVivo10 (QSR International) software.

### Ethics and Dissemination

#### Research Ethics Approval

All procedures are approved by the ethics committee of Alberta University and the Northern Alberta Clinical Trials Research Centre, Canada.

#### Incentives

All participants will receive a coffee shop gift card after completing the study. The value of the coffee shop gift card will be CAD $25 (US $20.49).

### Withdrawal From the Study

The participants and substitute decision makers can request to withdraw from the study at any time, either verbally or in writing. The participants will be able to withdraw from the study at any time before the group analysis is calculated. If a participant withdraws, their information will not be taken into account for analysis. In the event that a participant requests to have their data destroyed, the research team will honor this request by shredding and recycling the paper records and erasing any records stored on a computer hard drive using commercial software apps designed to remove all data from storage devices. However, once all the participants’ data have been analyzed, a participant cannot withdraw. The participants will be informed of this in the consent letter. The deadline for withdrawal will be once all the participants’ data have been collected and the data analysis is underway. This will be around the 16th month of the study.

### Consent or Assent

Signed consent will be obtained from all participants in the study. For those who are unable to give their informed consent, one of the therapist researchers (CH or DY) or another therapist involved in the recruitment process (JS or GD) will approach each potential participant and his or her substitute decision maker to provide information about the study. If these potential participants and their substitute decision makers give their consent, the substitute decision makers will sign the consent form, and we will seek the potential participants’ assent.

### Confidentiality

We will assign numerical codes to the participants instead of using their names or other identifiers. Only the study coordinator will have access to the master list where these codes are linked to the participants’ first names. With the exception of direct conversations with each participant, their names will not be used, only their numbers will be used. Hard copies of the consent forms, questionnaires, and study notes will be kept in a locked filing cabinet in a laboratory (Corbett Hall 1-45, Faculty of Rehabilitation Medicine, University of Alberta, Edmonton, Canada). All the deidentified electronic study documents will be encrypted and stored on a password-protected computer located in a laboratory (Corbett Hall 1-45, Faculty of Rehabilitation Medicine, University of Alberta, Edmonton, Canada).

### Access to Data

All the principal investigators will be given access to the cleaned data sets. The master list will be stored on a password-protected computer located in the principal investigator’s laboratory (Corbett Hall 1-45, Faculty of Rehabilitation Medicine, University of Alberta, Edmonton, Canada). Only the study coordinator will have access to this master list. The data will be retained for 5 years. There are no plans for future use of the data other than publishing them in peer-reviewed journals and at conferences. The data will not become part of a data repository and will not be involved in the creation of a research database or registry for future research use. After 5 years, the data will be destroyed. This will be done by shredding the paper records. Records stored on a computer hard drive will be erased using commercial software apps designed to remove all data from storage devices.

### Quality Assurance and Safety

We have established an advisory committee to monitor the progress of the study and, if necessary, to provide recommendations to the team members for discontinuation, modification, or continuation of the study. This committee will include our current partners, as follows: (1) Glenrose Rehabilitation Hospital, (2) Royal Alexandra Hospital, (3) experts in the areas of economics and commercialization and experts in health technology assessment, (4) patient representatives who have undergone hand therapy, and (5) certified hand therapists.

We will follow the CONSORT guidelines for clinical trial feasibility [17]. In addition, we will assess the quality of our study using the PEDro (Physiotherapy Evidence Database) scale [24].

## Results

The FEPSim study was launched in April 2020. Currently, this study is on hold because of the global COVID-19 pandemic. The recruitment process is expected to resume by September 2020, and the primary impact analysis is expected to be conducted by December 2020.

The results of this project will inform the development of best practices for clients with clinical conditions of the forearm, wrist, and hand, which impact the health of more than 35,072 Albertans annually. Improved rehabilitation can decrease the time needed to achieve functional outcomes, thereby decreasing health care costs. Returning patients more quickly to their valued occupations, including work, can decrease the costs associated with home care and other social services and reduce injury-related work time loss. FEPSim takes up little space, is much less expensive than comparable devices, and can easily be implemented throughout the public and private sectors in Alberta’s health system. This project is an excellent example of how industries and the health care system can support each other to grow and diversify Alberta’s economy and promote the entry of this valuable technology into the global rehabilitation market.

## Discussion

The primary objective of this study is to assess the feasibility of conducting a definitive trial on the effectiveness and cost-effectiveness of the FEPSim device for individuals with medical conditions that affect hand function. The level of evidence for the rehabilitation of hand function due to MSDs and neurological injuries and diseases is conflicting and differs according to the medical condition as well as the strategies or modalities implemented and the equipment and materials used during the sessions. The equipment that is currently available for hand therapy is either low cost or does not allow therapists to grade their therapeutic activities and exercises accurately, or it consists of high-tech devices that many clinicians or health care systems cannot afford. FEPSim has the potential to become a sound alternative in the midpoint between these 2 extremes. FEPSim has a technological readiness level of 7 [11]; thus, it has a sufficient level of readiness that can be tested in a real-world clinical setting. This feasibility study is the first RCT to evaluate the potential benefits of the FEPSim, not only in terms of functional outcome variables but also in terms of the costs associated with the delivery of hand rehabilitation in 2 large hospitals. Conducting this RCT will provide valuable information. First, the estimates can be used for sample size calculations in future RCTs. Second, as we will measure the patients’ outcome variables on 4 different occasions during the intervention, the results of this study will guide therapists by providing the expected percentage of a patient’s improvement and data on how the progress is made over a period of 10 weeks. The literature suggests that early hand therapy has an effect on range of motion and strength [12]; thus, this study will provide information about FEPSim’s potential to speed up the patient recovery process and reduce the length of treatment, which in turn may reduce treatment costs. In addition, the findings of this project can be used by therapists to develop exercise or activity standard protocols for hand rehabilitation interventions using the FEPSim device. Third, the estimations of the resource use and associated costs of the interventions during each arm of the trial will inform individuals who are responsible for purchasing or procurement decisions and who work with hospital budgets. The results of this project will provide them with information about the feasibility of adopting FEPSim.

In conclusion, this study will provide valuable information, such as the measurement of comparative intervention effects, technology acceptance by hand therapists, the associated costs of treatment, and product costs. This will contribute to the evidence planning process, which will be crucial for the future adoption of FEPSim.

## Abbreviations

AICE: Accelerating Innovation Into Care
AROM: active range of motion
BTE: Baltimore Therapeutic Equipment
CONSORT: Consolidated Standards of Reporting Trials
MSD: musculoskeletal disorder
PEDro: Physiotherapy Evidence Database
PROM: passive range of motion
PRWE: patient-rated wrist evaluation
RA: research assistant
RCT: randomized controlled trial
